# A Novel Minimally Invasive Surgical Technique for Eight-Plate Hemiepiphysiodesis: Description and Evaluation

**DOI:** 10.3390/jcm13175197

**Published:** 2024-09-02

**Authors:** Stephan Heisinger, Johannes Sommeregger, Carmen Trost, Madeleine Willegger, Markus Schreiner, Reinhard Windhager, Alexander Kolb

**Affiliations:** 1 Department of Orthopedics and Trauma Surgery, Medical University of Vienna, 1090 Vienna, Austria; 2 Teaching Center, Medical University of Vienna, 1090 Vienna, Austria

**Keywords:** minimally invasive surgical procedures, growth plate, genu valgum, genu varum

## Abstract

**Background:** Temporary hemiepiphysiodesis with tension band plates or eight-plates is a common surgical procedure to treat malalignment of the lower limb axis in skeletally immature patients. The objective of this study was to compare a new minimally invasive surgical procedure with the conventional procedure and evaluate its safety and effectiveness in order to reduce the risk of hypertrophic scarring, which may cause functional impairment as well as cosmetic issues. **Methods:** Sixty-five growth plates of either the femur or the tibia were evaluated in 33 patients treated for genu valgum or varum between 2010 and 2017. Each growth plate was considered an individual case. The modified procedure was used in 17 cases and the conventional procedure in 48 cases. The modified surgical procedure is characterized by an 8 mm incision and preparation of the epi-periosteal layer, in which the eight-plate is positioned via a guide-wire. Positioning and implantation are controlled via fluoroscopy. Skin incision length, duration of surgery, revision rate, achievement of a defined correction goal, and correction rate were analyzed. **Results:** Using the minimally invasive procedure, the mean skin incision length (23.94 ± 10.18 mm vs. 8.75 ± 2.14 mm, *p* < 0.001) could be significantly reduced. No significant difference was found in regard to the duration of surgery, revision rate, achievement of the correction goal or correction rate. **Conclusions:** The minimally invasive procedure results in a reduction in incision length without significant impact on the duration of surgery, revision rate, achievement of correction goal or correction rate. Consequently, the modified procedure can be regarded as equally as effective and safe as the conventional procedure.

## 1. Introduction

Malalignment about the knee, such as valgus or varus deformity, has been shown to consequently lead to an increased incidence of osteoarthritis in adulthood [1,2,3]. Generally, the lower limb axis changes from a slight varus to a slight valgus between the ages of 1 and 7 years; thereafter, however, a neutral alignment is expected [4,5,6,7]. Persisting lower limb deformities may lead to gait problems, pain, impaired functionality and potentially give rise to knee instability during childhood and adolescence [4,5,7]. As reviewed by Jelinek et al., consequently corrective osteotomies may be required in skeletally mature patients, while skeletally immature patients can be treated via less invasive procedures, such as temporary or permanent hemiepiphysiodesis [3,8]. While permanent hemiepiphysiodesis is definitive and thus requires exact planning and timing, temporary hemiepiphysiodesis is partially reversible due to residual growth after implant removal [3,9,10,11,12,13]. Nevertheless, temporary hemiepiphysiodesis also requires proper preoperative planning and timing using tools such as the Paley multiplier method, which is currently most commonly used [14,15]. Blount et al. established a procedure for temporary hemiepiphysiodesis via epiphyseal stapling; however, various implant-related complications have been reported [10,16,17,18,19]. In 2007, Stevens et al. established a novel method using tension band plates for angular correction, which has been shown to be associated with fewer implant-related complications and easier implantation by various authors [20,21,22]. These tension band plates are commonly referred to as eight-plates (eight-Plate^®^, Orthofix, McKinney, TX, USA). At our institution, we have developed a novel minimally invasive surgical technique for eight-plate implantation based on a modification of the surgical procedure described by Stevens et al. [22]. An obvious advantage of this novel surgical technique is the reduction of the length of the skin incision, resulting in a more favorable aesthetic outcome. However, another crucial aspect and potential complication of any surgical procedure is hypertrophic scarring and keloid formation in the proximity of a joint due to the mechanical forces on the wound [23]. Besides potential cosmetic issues that the patient may encounter due to hypertrophic scarring, it can also lead to functional impairment via contractures, specifically in the proximity of joints [24]. Considering that function-limiting deficits resulting from contractures due to excessive scarring can impair normal development in the pediatric population, this further highlights the relevance of approaches to reduce both the scar size and, consequently, the risk for significant hypertrophic scarring [23,25]. Concludingly, we hypothesize that our modified surgical approach not only results in more favorable cosmetic outcomes but also reduces the risk of functionally impairing hypertrophic scars or keloid formation. As reviewed by Braun et al., injury to small cutaneous nerves may also cause prolonged pain in patients after hemiepiphysiodesis using eight-plates, and this potential issue could be addressed by further reducing the incision length [26].

The aim of this study is to evaluate this minimally invasive procedure in regard to effectiveness and safety compared to the conventional implantation method described by Stevens et al. [22]. Concludingly, we hypothesize that our modified procedure is equally as effective and safe as the conventional method while offering advantages such as a more favorable cosmetic outcome and reduced risk of hypertrophic scarring or keloid formation, as well as a reduced risk of injuring small cutaneous nerves.

## 2. Surgical Technique, Patients and Methods, Statistics

### 2.1. Patients and Methods

The study was approved by the institutional review board of the Medical University of Vienna (Ethical vote: K-Nr. 1622/2016). All methods were carried out in accordance with relevant guidelines and regulations. All legal guardians gave their informed consent prior to the inclusion in this study. The screening of the database of the General Hospital of Vienna yielded 37 patients who underwent hemiepiphysiodesis between 2010 and 2017. Inclusion criteria were patients with idiopathic deformities who had adequate follow-up with physical and radiographic assessment before surgery and, at the latest, before implant removal. Inclusion criteria consisted of an adequate clinical and radiographic follow-up with physical evaluation and radiographic assessment before implantation of either device and at the time of removal [3]. Exclusion criteria were (1) lack of follow-up; (2) significant dysplasia of the hip and knee joint; (3) previous surgeries at the knee joint; and (4) a concomitant musculoskeletal disease. Concludingly, we retrospectively analyzed 33 patients who underwent hemiepiphysiodesis using 8-plates (eight-Plate^®^, Orthofix, McKinney, TX, USA), resulting in 65 cases of hemiepiphysiodesis (female: 49.2%; male: 50.8%). The mean age at surgery was 13.23 ± 1.77 years. In 48 (73.8%) cases, the conventional implantation procedure was performed, and in 17 (26.3%) cases, the minimally invasive procedure was performed. The majority of cases (58 cases; 89.2%) were treated for a valgus deformity, while the rest (7 cases; 10.8%) were treated for a varus deformity.

All radiographic measurements were performed by an orthopedic surgeon using TraumaCad (Voyant Health, Petach-Tikva, Israel, Version 2.5) on standardized anterior–posterior (AP) views of both lower extremities. Analogous to Jelinek et al., one image was taken shortly before implantation, and one image was taken up to 2 months prior to implant removal [3]. Analyses included measurements of mechanical axis deviation (MAD), mechanical lateral distal femoral angle (mLDFA) and mechanical medial proximal tibial angle (MPTA) [3,27]. Additionally, we determined the rate of correction (ROC) analogous to Danino et al. based on the changes of mLDFA for cases with 8-plate implantation at the distal femur and MPTA for cases with 8-plate implantation at the proximal tibia as °/month [28].

Furthermore, clinical parameters, such as correction period, operation time, length of skin incision, revision rate, achievement of correction goal and side effects, were recorded. The patients received clinical examination and conventional radiography controls on a 3-monthly basis after surgery by experienced pediatric orthopedic surgeons until implant removal. Our primary outcome variables were correction period, operation time, length of skin incision, revision rate, achievement of correction goal and occurrence of complications in order to determine the efficacy and safety of our modified technique.

### 2.2. Surgical Technique

**Figure 1:**
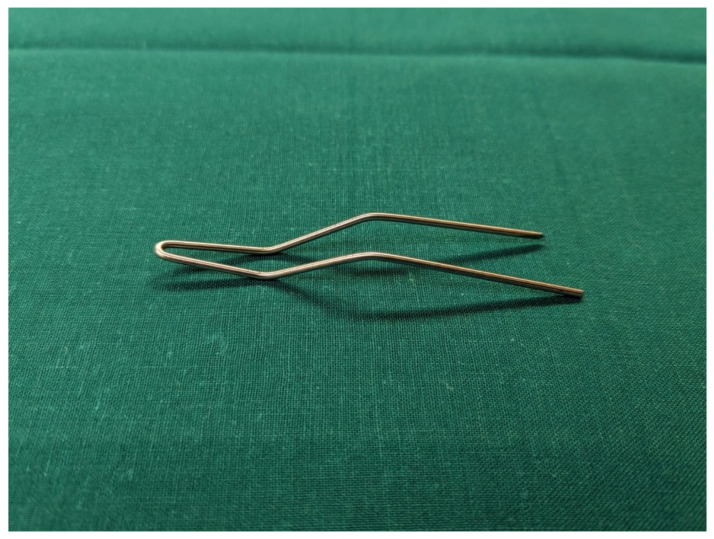
The K-wire bent into a U-shape with arms of equal length to create a guidance device.

**Figure 2:**
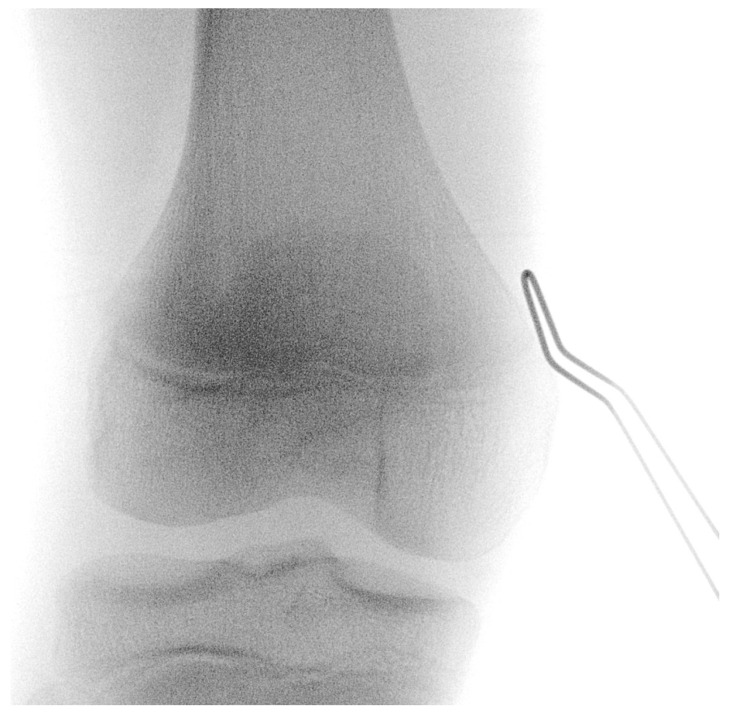
The modified K-wire is placed in the epi-periosteal layer—positioning is confirmed via fluoroscopy; the K-wire is used as a rail to ensure that the 8-plate is consistently placed in the desired epi-periosteal layer after insertion.

**Figure 3:**
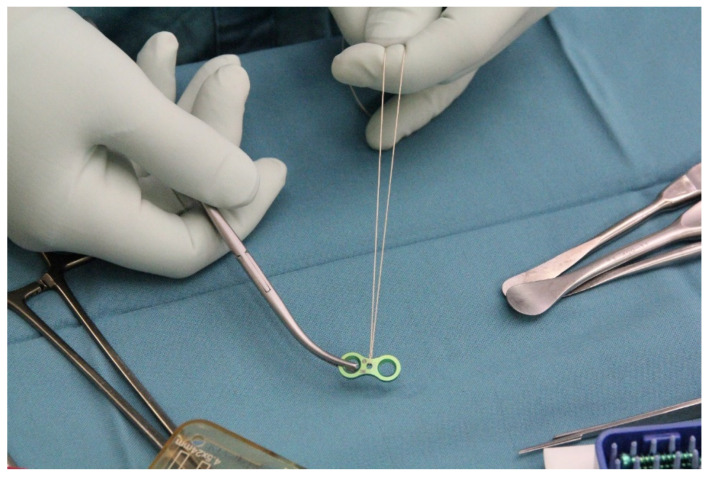
The 8-plate secured via suture to enable easy retrieval of the implant in case of displacement.

**Figure 4:**
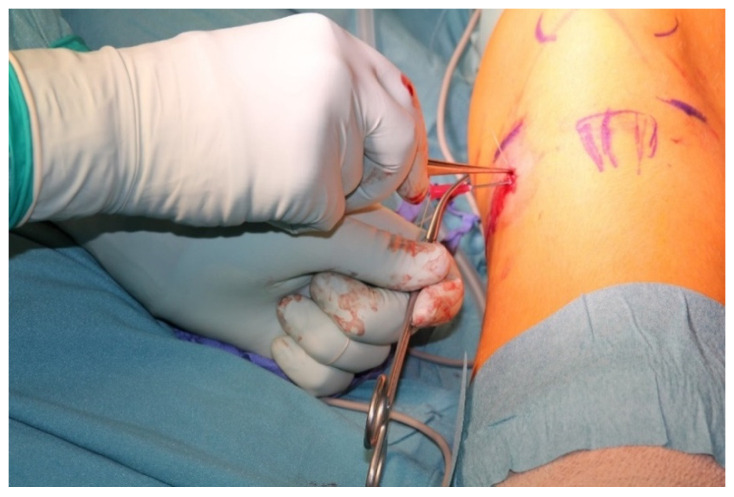
Positioning of the implant under fluoroscopy guidance.

**Figure 5:**
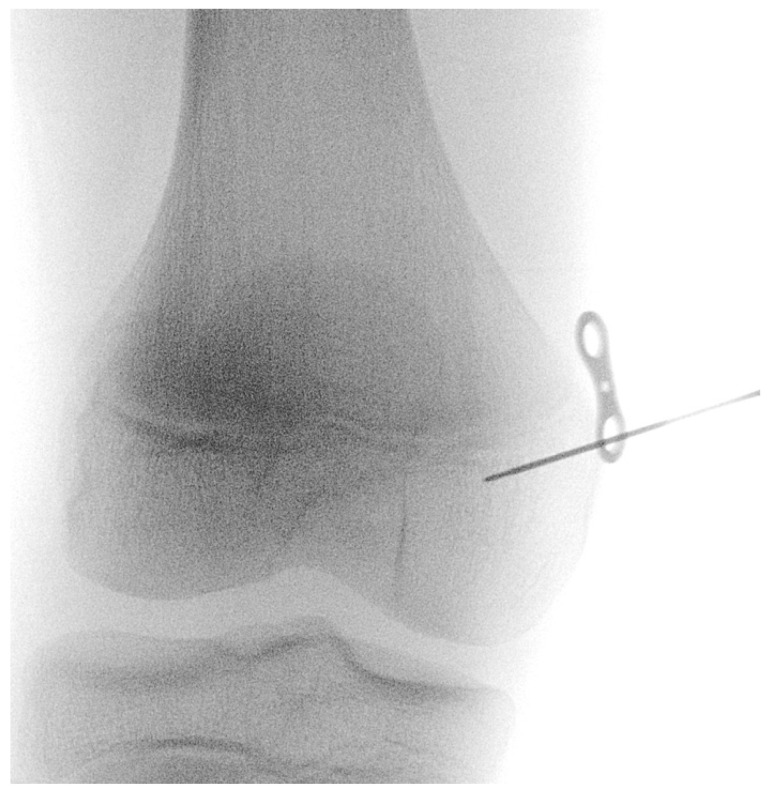
K-Wire is inserted into the epiphysis through the appropriate hole of the 8-plate.

**Figure 6:**
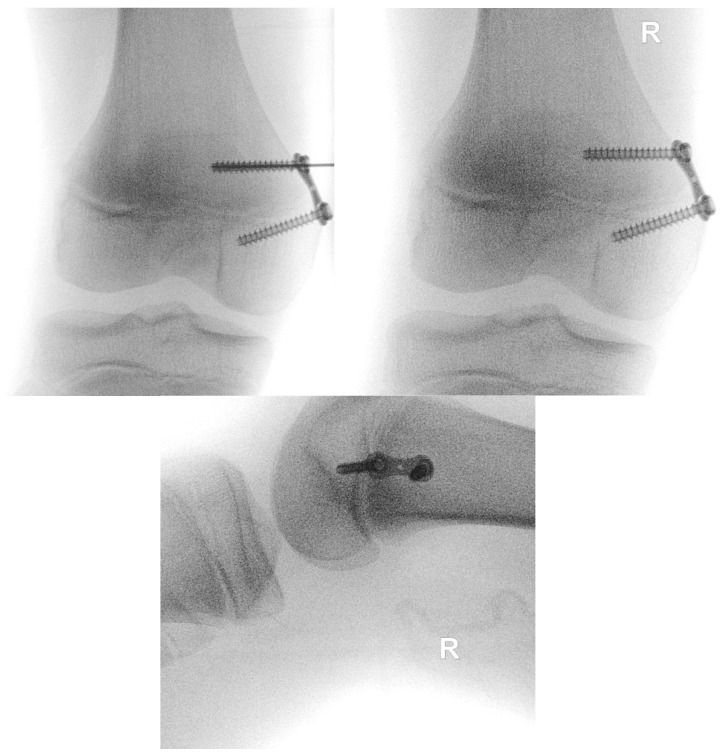
Subsequent screw insertion under fluoroscopy guidance.

**Figure 7:**
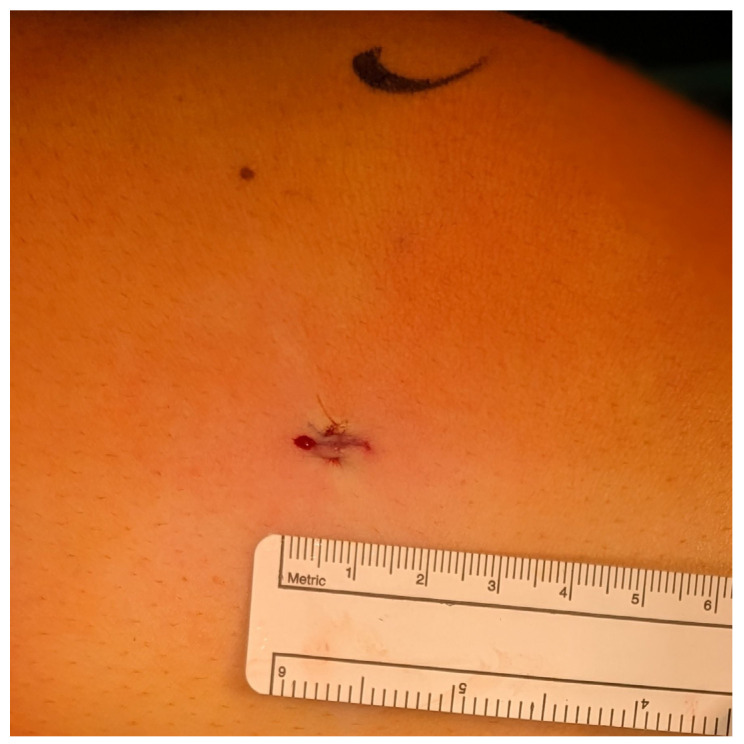
Intracutaneous suture—appr. 8 mm incision length.

The procedure is performed under general anesthesia and the patient is placed in a supine position on the operating table. After surgical washing and draping, the epiphysis is located using fluoroscopy. Accordingly, a surgical incision of approximately 8 mm is made, and further blunt dissection is performed until an epi-periosteal layer can be identified. A K-wire is then bent into a U-shape with arms of equal length (see Figure 1). The blunt end of the U-shaped K-wire is then placed in the epi-periosteal layer, and the epi-periosteal position is confirmed via fluoroscopy (see Figure 2). In the next step, the modified K-wire is used as a rail under which the 8-plate is inserted, ensuring that the 8-plate is consistently placed in the desired epi-periosteal layer. The placement of the implant in the correct layer is a crucial step of our procedure and, if not carried out with diligent care, may result in complications, such as soft-tissue interposition and, potentially, subsequent hardware failure or prolonged postoperative pain. The plate is secured with a suture during insertion so that it can be easily retrieved if it is misplaced (see Figure 3). This is essential considering the reduced incision length and subsequent difficulties in retrieving the implant in case of displacement. When the position of the plate in the correct layer is confirmed under fluoroscopy, the K-wire is removed (see Figure 4). A new K-Wire is then inserted into the epiphysis through the appropriate hole of the 8-plate under fluoroscopic guidance (see Figure 5). Hereafter, the first cannulated screw is placed over this wire. When the K-wire is removed again, another K-wire is inserted into the metaphysis through the other hole of the 8-plate, and the second screw is placed over this wire (see Figure 6). Fluoroscopic guidance is a key element of the procedure, as the plate cannot be seen directly during the procedure. Finally, the wound is closed using an intracutaneous suture (see Figure 7). The same incision will be used for implant removal after reaching the correction goal.

### 2.3. Statistics

Data were stored and processed for further analysis in MS Excel (Microsoft Corporation, 2018. Microsoft Excel, available at: https://office.microsoft.com/excel, accessed on 6 June 2020). Statistical analysis was carried out using SPSS (IBM Corp. Released 2020. IBM SPSS Statistics for Windows, Version 27.0. IBM Corp., Armonk, NY, USA). For metric data analysis, mean, standard deviation, median, minimum and maximum were determined. Absolute and relative frequencies were determined for the nominal parameters. The samples were tested for normality using Shapiro–Wilk tests. To identify a statistically significant difference, *t*-tests for independent samples, Mann–Whitney U-test and Fisher’s exact test were applied. An alpha of 0.05 was assumed to constitute statistical significance.

## 3. Results

In the minimally invasive study group (group 1), 11 cases (64.7%) were female and 6 cases (35.3%) were male, with a mean age at surgery of 12.66 ± 2.22 years. Hemiepiphysiodesis was performed at the distal femur in nine cases (52.9%) and at the proximal tibia in eight cases (47.1%). All cases in group 1 were treated for valgus deformity. All patients in group 1 were operated on by a single experienced orthopedic surgeon, and patients in the conventional group were operated on by multiple surgeons. Overall, one patient was excluded due to lack of follow-up, one patient was excluded due to significant dysplasia of the hip and knee joint and one patient was excluded due to numerous previous surgeries at the knee joint. Another patient was excluded due to a concomitant musculoskeletal disease, resulting in 65 cases of hemiepiphysiodesis.

In the conventional study group (group 2), 21 cases (43.8%) were female, and 27 cases (56.3%) were male, with a mean age at surgery of 13.43 ± 1.56 years. In this group, hemipepiphysiodesis was performed at the distal femur in 28 cases (58.3%) and at the proximal tibia in 20 cases (41.7%). In total, 41 cases (85.4%) were treated for valgus deformity, and 7 cases (14.6%) were treated for varus deformity.

The patients in group 1 (12.66 ± 2.22 years) were slightly younger than in group 2 (13.43 ± 1.56); normality was found in group 1 but not in group 2 using the Shapiro–Wilk test. However, no significant difference was found using the Mann–Whitney U-test (U = 345.0; *p* = 0.351). In group 1 (*n* = 17), one revision surgery (5.9%) was performed due to an excessive rebound effect; in group 2 (n = 48), revision surgery was performed in two cases (4.2%). In one case, revision surgery was performed due to an excessive rebound effect, and in the other case, correction failed to appear at all. Overall, no significant difference was detected between the two groups (*p* = 1.000). Otherwise, no complications were observed.

The length of the skin incision was documented in 28 cases. In group 1 (n = 12), the mean length was 8.75 ± 2.14 mm; in group 2 (n = 16), the mean length was 23.94 ± 10.18 mm. The Mann–Whitney U-test showed a significant difference between the two groups (U = 2.5; *p* < 0.001).

**Figure 8:**
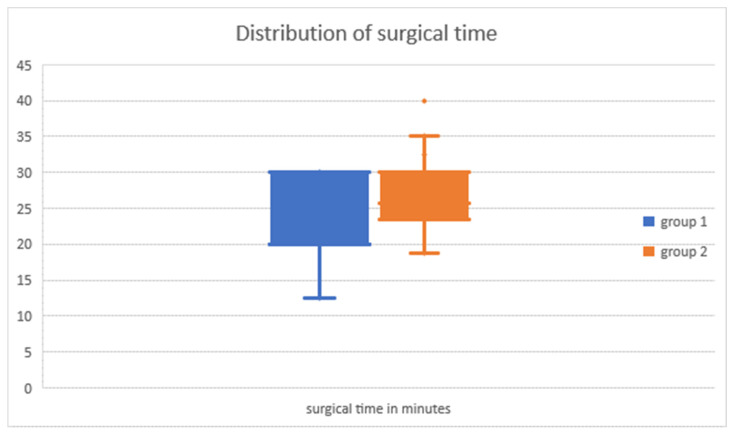
Duration of surgery in minutes: group 1 (minimally invasive) and group 2 (conventional).

In 10 cases, the surgical time could not be determined; in one patient, both surgical procedures were performed, but the exact duration was not recorded for every single procedure. In another eight cases, additional procedures were performed besides hemiepiphysiodesis. The mean surgical time in group 1 (n = 12) was 22.27 ± 6.37 min and in group 2 (n = 34), 26.47 ± 5.89 min; however, this difference was not statistically significant (*p* = 0.160; see Figure 8).

**Figure 9:**
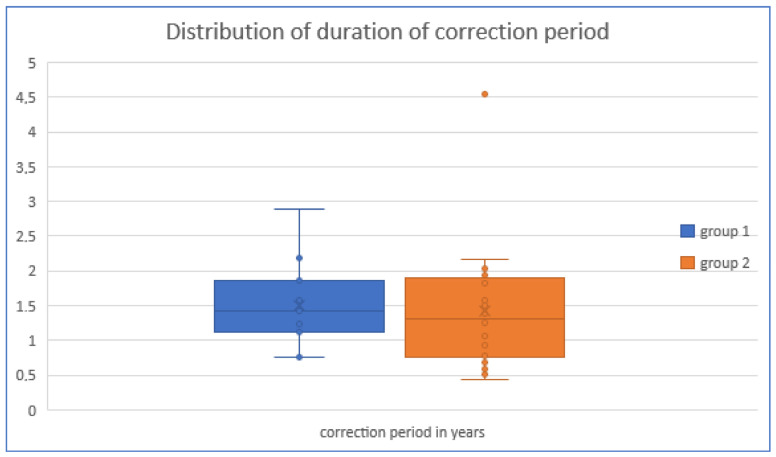
Duration of correction period: no significant difference between group 1 (minimally invasive) and group 2 (conventional) was found (U = 395.5; *p* = 0.322).

In five cases, no correction period could be determined because no hardware removal was performed. In group 1 (n = 15), the mean correction period was 1.50 ± 0.61 years, and in group 2 (n = 45), 1.42 ± 0.88 years. No significant difference was found between the two groups (U = 395.5; *p* = 0.322). In five cases, no correction period could be determined because no hardware removal was performed (see Figure 9).

The correction goal in group 1 (n = 17) was reached in 12 cases (70.6%); in group 2 (n = 48), it was reached in 40 cases (83.3%). No significant difference was found between both groups (Fisher’s exact test; *p* = 0.299).

**Figure 10:**
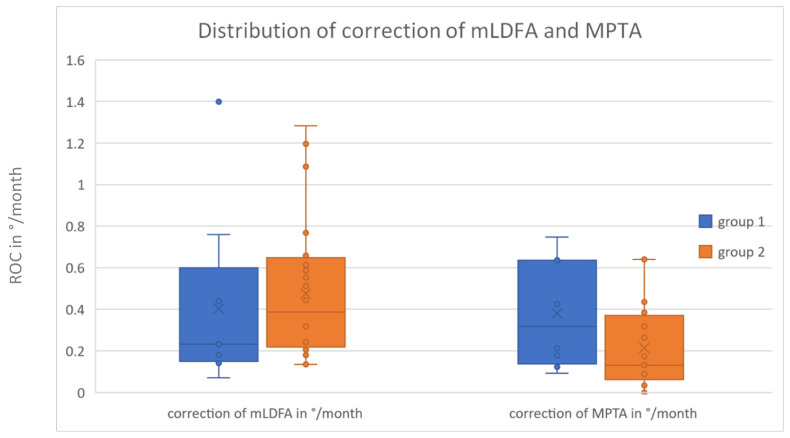
ROCs of mLDFA and MPTA: group 1 (minimally invasive) and group 2 (conventional).

In 37 cases, hemiepiphysiodesis was performed at the distal femur. The mean rate of correction (ROC) of the mLDFA in group 1 (n = 9) was 0.40 ± 0.43 °/month and 0.48 ± 0.32 °/month in group 2 (n = 28) (see Figure 10).

In 28 cases, hemiepiphysiodesis was performed at the proximal tibia. The ROC in group 1 (n = 8) was 0.38 ± 0.26 °/month and in group 2 (n = 20), 0.21 ± 0.20 °/month.

The difference in the ROCs of the distal femur and proximal tibia between the two groups was not statistically significant (*p* = 0.240 and *p* = 0.089; see Figure 10).

## 4. Discussion

**Figure 11:**
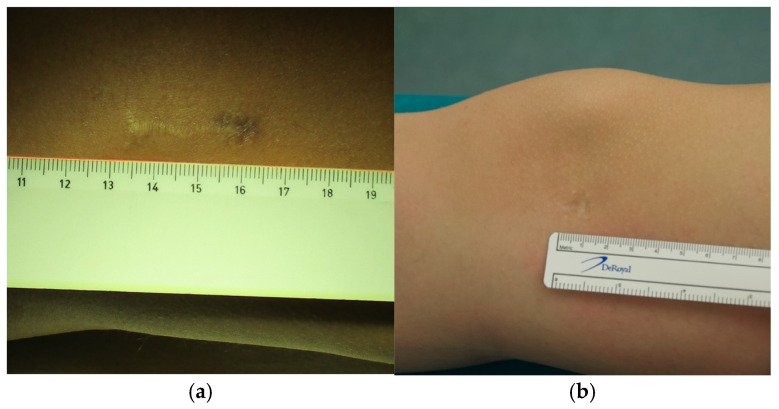
Scar formation following (**a**) conventional and (**b**) minimally invasive procedures.

**Figure 12:**
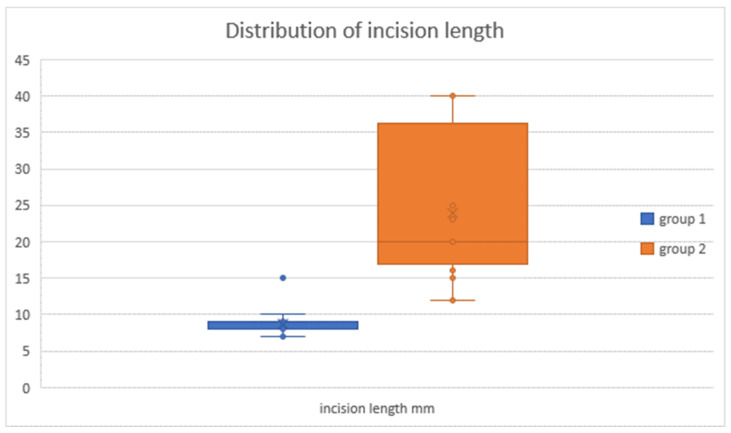
*Skin incision length:* in group 1 (minimally invasive), the mean skin incision length was 8.8 ± 2.1 mm; in group 2 (conventional), the mean skin incision length was 23.9 ± 10.2 mm.

We describe a novel minimally invasive surgical technique for temporary hemiepiphysiodesis using tension band plates, which is a modification of the well-established procedure for deformity correction around the knee described by Stevens et al., which has been shown to be effective and safe by various authors [3,22,29]. The minimally invasive surgical procedure described here reduces the length of the skin incision, which leads to a more favorable aesthetic result. As shown in Figure 11, the scar is almost undetectable a few months after the surgery, highlighting the advantage of our modified technique in this regard compared to previously described procedures (see Figure 11) [22,30]. This further reduction in incision size is the main advantage of our minimally invasive procedure (see Figure 12); however, we believe that it demands a higher surgical skill level compared to conventional procedures. As depicted in Figure 11b, the scar in patients who underwent the minimally invasive procedure is barely visible, and even in the case of hypertrophic scarring, it would not lead to functionally impairing contractures. On the other hand, a wound length of up to 3 cm in close proximity to a joint is proportionally large in pediatric patients and may lead to functionally relevant scarring in case of keloid formation. Taking these considerations into account, this further highlights the relevance and validity of our modified surgical approach.

Potential pitfalls may be the malpositioning of the implant due to soft-tissue interference or, potentially, the partial entrapment of the iliotibial tract beneath the plate if the correct layer is not identified according to our described procedure. The malpositioning of the implant may consequently cause pain, implant failure and lack of correction. Therefore, the use of the U-shaped K-wire as a guidance device is crucial for our modified technique to practically eliminate the risk of malpositioning. Due to the aforementioned precautions, no malpositioned implant was detected in our study population. Nevertheless, this may be considered a limitation of the novel procedure to a certain degree.

We have found the duration of surgery (group 1: 22.3 ± 6.4 min; group 2: 26.5 ± 5.9 min, see Figure 8) to be comparable to those reported, e.g., by Masquijo et al. [30]. This may be a peculiar finding considering the complex nature of our modified technique with additional steps, such as the application of the U-bent K-wire to ensure positioning in the correct layer. However, by applying standardized control mechanisms, we were able to improve the overall workflow and reduce the need for repeated verification of the positioning of the implant in the correct layer. Interestingly, the ROCs we have observed in our study were slightly lower than those reported by various authors, which may be attributed to the younger age at surgery in other studies [20,28,31].

As reviewed by Krakowski et al., we must not forget to consider the potential psychosocial distress that a pediatric patient may experience, which does not necessarily correlate with the clinician-rated severity of the scar [24]. Although the knee joint might not be a particularly stigmatizing location for a scar, regardless, it may cause psychological distress in a pediatric patient in case of hypertrophic scarring or keloid formation. Taking this aspect into account, the seemingly trivial advantage of a smaller scar turns out to be significantly more relevant than one might expect at first.

As stated by Gupta et al., injury to cutaneous nerves may cause prolonged pain in patients who received hemiepiphysiodesis via tension band plating; consequently, this further highlights another advantage of our modified surgical procedure via the reduction of incision length [7].

The main limitation of our study is the retrospective study design and the relatively low sample size. Nevertheless, we were able to show that our modified technique is equally effective and safe compared to the conventional technique. While delivering equally good outcomes, the application of our modified technique results in a scar that is barely detectable and consequently additionally reduces the risk of potential functional impairments as well as physical, psychological and social comorbidities in case of hypertrophic scarring or keloid formation [24].

## 5. Conclusions

We have described a novel minimally invasive surgical technique for hemiepiphysiodesis via eight-plates, which is a modification of the technique described by Stevens et al. [22]. Our proposed surgical technique can be used to reduce the size of the incision, and consequently, it not only yields a more favorable cosmetic outcome but also reduces the risk of functional impairments and other comorbidities in case of hypertrophic scarring. Moreover, when caring for pediatric patients, scars can cause psychosociological distress that does not necessarily correlate with objective scar severity; hence, this highlights the relevance of a reduction in incision length. Furthermore, the reduced incision length also reduces the risk of injuries to cutaneous nerves. Altogether, we were able to show that our modified surgical technique can be considered a safe and effective method for guided growth with the above-mentioned advantages. Concludingly, we have established a novel surgical technique that has the potential to crucially improve patient care and patient satisfaction. However, future studies with a prospective design and a larger patient sample will be needed to further validate our novel surgical technique.

## Data Availability

The raw data supporting the conclusions of this article will be made available by the authors on request.
